# Supplementary material for: MetaRibo-Seq measures translation in microbiomes
Source: Nat Commun. 2020 Jun 29;11:3268. doi: 10.1038/s41467-020-17081-z (PMC7324362; doi:10.1038/s41467-020-17081-z)
Supplement: Supplementary file 10 — Supplementary Data 7 [file 41467_2020_17081_MOESM10_ESM.zip › File2/Confidence_High_Taxonomy/262846_out.krona.html]

Javascript must be enabled to view this page.

members
magnitude
magnitudeUnassigned
count
unassigned
taxon
rank

262846\_out

7

superkingdom
7
2

7
1239
phylum

7
186801
class

order
7
186802

family
4
186803

genus
2569097
4

species

SRS015431\_contig\_number\_80191SRS017521\_contig\_number\_38587SRS143342\_contig\_number\_20220SRS148159\_contig\_number\_8299
39488
4

186806
3
family

1730
3
genus
1

SRS144183\_contig\_number\_39150

2
165185

SRS077502\_contig\_number\_4064SRS148721\_contig\_number\_38514
species
